# The complete chloroplast genome of *Vicia cracca* L

**DOI:** 10.1080/23802359.2021.1926355

**Published:** 2021-06-14

**Authors:** Yupeng Guo, Jiali Ma, Junqiao Li

**Affiliations:** Qinghai Provincial Key Laboratory of High value Utilization of Characteristic Economic Plants, College of Ecological Environment and Resources, Qinghai Nationalities University, Xining, P. R. China

**Keywords:** *Vicia cracca* L, chloroplast genome, phylogenetic analysis

## Abstract

*Vicia cracca L*. is a widespread perennial herb in the Northern Hemisphere. It has purple flowers and leave tendrils for climbing on neighboring vegetation. For knowing the chloroplast genome, a sample’s genomic was extracted, sequenced, assembled and annotated. The chloroplast genome of this plant is a circular form of 126,272bp in length with IR loss. After annotation, a total of 108 genes were predicted, of which, 75 encode proteins, 3 rRNA, 30 tRNA. The evolutionary history, inferred using Maximum Likelihood method, indicates that *V. cracca* was grouped within *Vicia* in Fabaceae. The complete cp genome will be helpful for further studies on molecular biology, evolution, population genetics, taxonomy or resources protection.

*Vicia cracca* L., belonging to Fabaceae, is widespread in the Northern Hemisphere. It is a polycarpic perennial herb and often found in meadows, along roads, river banks, and forest margins. This plant has dense pedunculate racemes inflorescences comprising of 10–30 purple flowers and with leave tendrils, its stems can climb on neighboring vegetation (Eliášová et al. 2014; Eliášová and Münzbergová 2017). In previous studies, many literatures focused on chemical composition, genetic diversity of autotetraploids in natural populations, infecting viruses, root nodulating *Rhizobium leguminosarum* (Christian et al. 1982; Eliášová et al. 2014; Van Cauwenberghe et al. 2014; Gallet et al. 2018). In this study, we report the complete chloroplast (cp) genome of *V*. *cracca*., and analyzed the relationship with other related species by phylogenetic analysis.

Samples from Qilian mountains (36°34′37″N,101°48′27″E) in Qinghai province were collected for sequencing. A specimen was deposited at College of Ecological Environment and Resources, Qinghai Nationalities University (https://shxy.qhmu.edu.cn/, Junqiao Li, email: ljqlily2002@126.com) under the voucher number HCEERQNU-20200827001. A sample’s total genomic DNA was extracted from about 100 mg fresh leaves using a modified CTAB method (Murray and Thompson 1980). Paired-end Libraries with an average length of 350 bp were constructed and sequenced on the Illumina Novaseq platform (Shenzhen Huitong Biotechnology Co. Ltd). In total, 13,677,281 raw reads were obtained with 350X coverage. After filtered by NGSQCToolkit v2.3.3 (Patel and Jain 2012), 13,625,886 clean reads were used for assembling with the de novo assembler SPAdes (Bankevich et al. 2012). Gene annotation was performed via PGA (Qu et al. 2019).

The complete cp genome of *V. cracca* (GenBank accession no. MW266076) is a circular form of 126,272bp in length with 34.8% GC content and IR loss which is common in Fabaceae, especially in Papilionoideae (Cai et al. 2008; Yi et al. 2020). A total of 108 genes were predicted on this cp genome to composing of 75 encode proteins, 3 rRNA and 30 tRNA.

Phylogenetic analysis was performed based on complete cp genomes of *V. cracca* and other 27 related species reported in Fabaceae, two species in Polygalaceae as outgroup. The genome-wide alignment of 30 genomes was constructed by HomBlocks (Bi et al. 2018). The evolutionary history was inferred with Maximum Likelihood (ML) method by IQ-TREE 1.6.12 under TVM + F+R3 model (Nguyen et al. 2015; Kalyaanamoorthy et al. 2017), and the output file was edited in MEG X (Kumar et al. 2018). Bootstrap (BS) values were calculated with UFBoot2 from 1000 replicates (Hoang et al. 2018). As expected, *V. cracca* was grouped within *vicia* in Fabaceae (Figure 1). The phylogenetic relationship was generally consistent with the result of Schaefer’s research on tribe Fabeae Systematics (Schaefer et al. 2012). The complete cp genome of *V. cracca* will be helpful for further studies on molecular biology, evolution, population genetics, taxonomy or resources protection.

**Figure 1.**
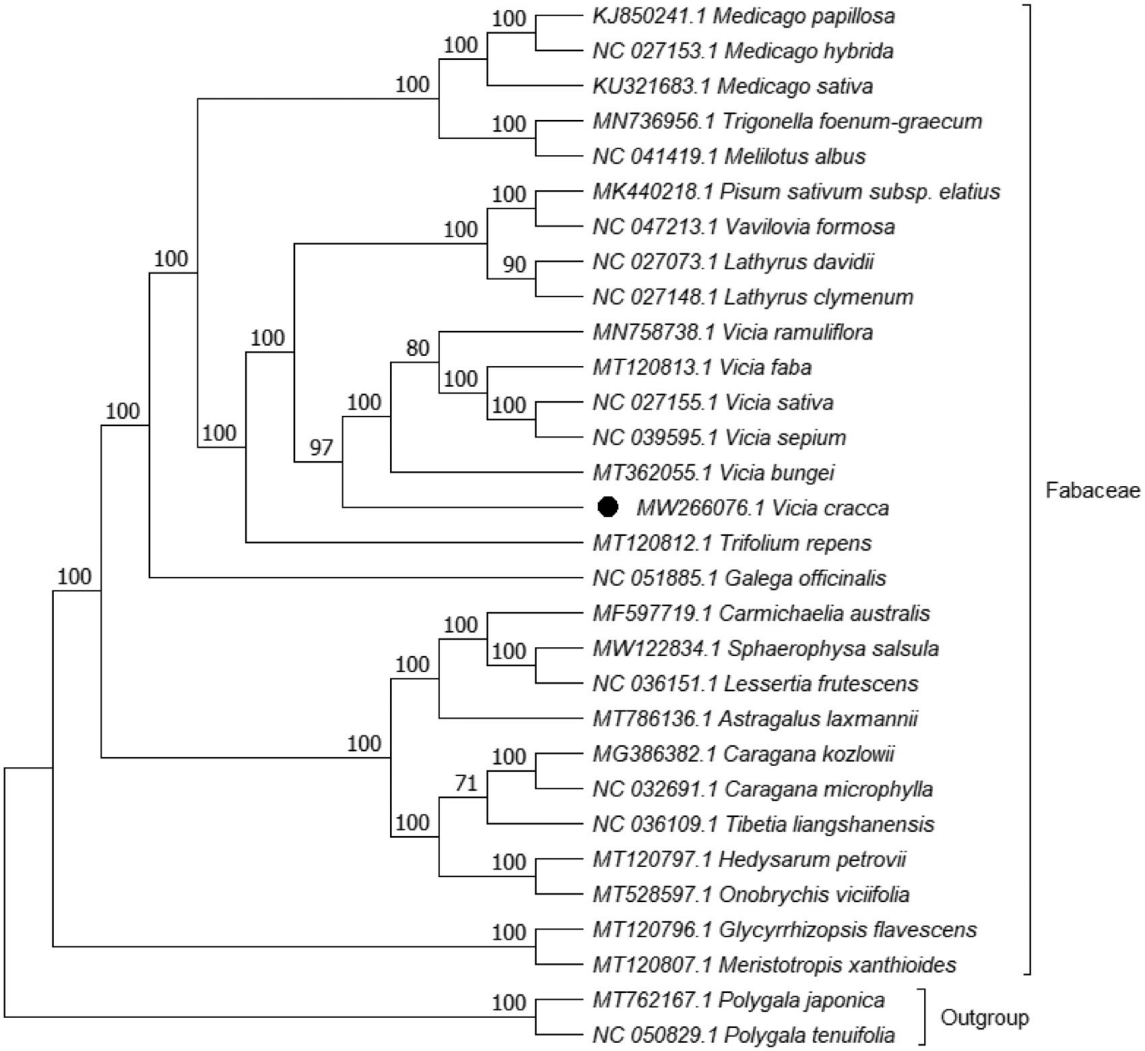
ML phylogenetic tree based on 30 species chloroplast genomes was constructed using IQ-TREE 1.6.12. Numbers on each node are bootstrap support values from 1000 replicates.

## Data Availability

The genome sequence data that support the findings of this study are openly available in GenBank of NCBI at (https://www.ncbi.nlm.nih.gov/nuccore/MW266076) under the accession no. MW266076. The associated BioProject, SRA, and Bio-Sample numbers are PRJNA687614, SRR13300064, and SAMN17150903, respectively.
